# Use of thymidine analogues to indicate vascular perfusion in tumours

**DOI:** 10.1054/bjoc.2000.1372

**Published:** 2000-09-04

**Authors:** A C Begg, I Hofland, I Van Der Pavert, B Van Der Schueren, K Haustermans

**Affiliations:** Division of Experimental Therapy, Department of Biophysics, The Netherlands Cancer Institute, Plesmanlaan 121, Amsterdam, CX 1066, The Netherlands; Department of Radiotherapy, University Hospital, Gasthuisberg, Leuven Belgium

**Keywords:** tumour perfusion, IdUrd, hypoxia, image analysis, flow cytometry

## Abstract

Temporary reduction in blood-flow within tumour blood vessels can reduce oxygen supply leading to transient perfusion-limited hypoxia. Consequent selection of cells with mutations and reduced radiosensitivity can lead to disease progression and treatment-resistance. In the present study, we investigated whether heterogeneity of labelling after thymidine analogue administration is related to perfusion variations, and if so, could it be quantified and used as a perfusion indicator. Perfusion in murine RIF1 tumours was reduced by hydralazine or increased by nicotinamide and the mice subsequently injected with IdUrd. Tumours were halved for analysis by both flow cytometry and immunohistochemistry. Tumour sections were stained for vasculature and IdUrd. Each blood vessel was scored for the density of IdUrd-labelled cells surrounding it, using a semi-quantitative scoring system. Flow cytometry showed that the IdUrd labelling index and intensity decreased by approximately 50% after hydralazine. In tumour sections of control animals, 2.9% of vessels showed no IdUrd label. In contrast, after hydralazine almost 50% of vessels had no surrounding IdUrd labelling, whereas after nicotinamide there were fewer vessels with low labelling and a higher median score. In conclusion, changes of tumour perfusion by pharmacological agents is reflected in changes in tumour-cell labelling by the thymidine analogue IdUrd, suggesting that IdUrd labelling could be used to indicate perfusion in individual vessels in human tumours. © 2000 Cancer Research Campaign


British Journal of Cancer (2000) **83**(7), 899–905
© 2000 Cancer Research Campaign
doi: 10.1054/ bjoc.2000.1372, available online at http://www.idealibrary.com on IDEAL®

### Use of thymidine analogues to indicate vascular perfusion in tumours

**AC Begg[1], I Hofland[1], I Van Der Pavert[2], B Van Der Schueren[3] and K Haustermans[1,3]**

[1]Division of Experimental Therapy; [2]Department of Biophysics, The Netherlands Cancer Institute, Plesmanlaan 121, 1066 CX Amsterdam, The Netherlands; [3]Department of Radiotherapy, University Hospital, Gasthuisberg, Leuven, Belgium.

**Summary** Temporary reduction in blood-flow within tumour blood vessels can reduce oxygen supply leading to transient perfusion-limited hypoxia. Consequent selection of cells with mutations and reduced radiosensitivity can lead to disease progression and treatment-resistance. In the present study, we investigated whether heterogeneity of labelling after thymidine analogue administration is related to perfusion variations, and if so, could it be quantified and used as a perfusion indicator. Perfusion in murine RIF1 tumours was reduced by hydralazine or increased by nicotinamide and the mice subsequently injected with IdUrd. Tumours were halved for analysis by both flow cytometry and immunohistochemistry. Tumour sections were stained for vasculature and IdUrd. Each blood vessel was scored for the density of IdUrd-labelled cells surrounding it, using a semi-quantitative scoring system. Flow cytometry showed that the IdUrd labelling index and intensity decreased by approximately 50% after hydralazine. In tumour sections of control animals, 2.9% of vessels showed no IdUrd label. In contrast, after hydralazine almost 50% of vessels had no surrounding IdUrd labelling, whereas after nicotinamide there were fewer vessels with low labelling and a higher median score. In conclusion, changes of tumour perfusion by pharmacological agents is reflected in changes in tumour-cell labelling by the thymidine analogue IdUrd, suggesting that IdUrd labelling could be used to indicate perfusion in individual vessels in human tumours. © 2000 Cancer Research Campaign

**Keywords**: tumour perfusion; IdUrd; hypoxia; image analysis; flow cytometry

Hypoxic cells in animal and human tumours are thought to arise primarily through two distinct mechanisms. The first is termed chronic, or diffusion-limited, hypoxia. This mechanism was first proposed by Thomlinson and Gray (1955) who observed viable cells around regions of oxygen supply in lung tumours, and necrosis at distances of around 150 μm consistent with the limit of oxygen diffusion combined with oxygen consumption rates in tumour tissue. The second mechanism is through transient changes in blood-flow (Sutherland and Franko, 1980; Chaplin et al, 1986; Brown, 1979). With this acute form of hypoxia, the affected cells may have a greater chance of viability because hypoxia is shorter and temporary. This could pose problems since hypoxia-mediated selection of cells with diminished apoptotic potential could occur, possibly accounting for resistance to radiotherapy and chemotherapy-induced apoptosis (Chaplin et al, 1986; 1987). Chronically, hypoxic cells are often energy-deficient and consequently less capable of DNA repair, rendering them sensitive to radiation (Durand, 1983; Spiro et al, 1984), and by inference, to other DNA-damaging agents. This would reduce their impact in determining the outcome of therapy. Acutely hypoxic cells, however, due to their transient nature, may be less nutrient- and energy-depleted, well capable of repair, and therefore resistant to therapy, both because of their hypoxic state (radiation) and their temporary removal from drug supply (chemotherapy). This emphasizes the need to include these cells in measurements of total hypoxia in human tumours.

*Received 9 August 1999*
*Revised 25 May 2000*
*Accepted 4 June 2000*

*Correspondence to:* AC Begg

There is now a large body of evidence in animal tumours showing that blood-flow in tumour vessels is disordered, shows wide variations in flow rate, and can temporarily or permanently cease, or even reverse in direction (Brown, 1979; Reinhold and van den Berg-Blok, 1983; Chaplin et al, 1986). This has been shown with a variety of techniques including implanted 'sandwich' chambers, injecting two intravascular markers separated in time (the mismatch technique) (Chaplin et al, 1987; Trotter et al, 1989*b*) and by using perfusion markers such as the Hoechst dye H33342 together with immunostaining for vessels (Bernsen et al, 1995; van Geel et al, 1996). The fraction of perfused vessels in animal tumours is often considerably less than 100%. Temporary flow reduction or cessation will almost certainly lead to a transient increase in hypoxia for those tumour cells supplied by the vessel (Kimura et al, 1996; Dewhirst, 1998).

Several markers are available for measuring vascular perfusion in animal tumours, including labelled dextrans, albumins, and fluorescent dyes such as Hoechst 33342 and DiOC7 (Trotter et al, 1989*b*). The marker is injected intravenously and a sample taken within minutes. High-molecular-weight markers (albumins and dextrans) are found largely intravascularly at these short sampling times, while Hoechst dyes diffuse out of vessels but bind in DNA of cells surrounding a vessel, thereby delineating areas of perfusion. Most of these are not suitable for human use for toxicity reasons, although labelled dextrans have been used with local administration (Huber et al, 1984; Bollinger, 1993).

Thymidine analogues such as iodo- and bromo-deoxyuridine (IdUrd, BrUrd) could be useful as indirect perfusion indicators. Both analogues are approved for clinical use and have undergone widespread testing and application as kinetic markers in human cancer (Begg, 1995; Begg et al, 1999). The presence and intensity of labelling, however, will depend not just on whether a cell is undergoing DNA synthesis, but also on analogue supply. In histological sections of both animal and human tumours, areas of light or even no labelling are not uncommon. These areas usually contain cells appearing normal and viable, and which will sometimes stain positively for intrinsic proliferation markers like histone H3 (Kotelnikov et al, 1997). A plausible explanation is that blood-flow is temporarily reduced in these areas, reducing or eliminating supply of the thymidine analogue. In the present study, we have investigated whether variations in IdUrd labelling can indirectly assess blood-vessel function. The advantage of using thymidine analogues is that they are already used on a wide scale in the clinic at non-toxic doses for cell kinetic studies, obviating the need for new drug development.

### MATERIALS AND METHODS

### Tumour model

Female C3H/Km mice, weighing 20–30 g, were used throughout. The RIF1 sarcoma was maintained according to recommended procedures of Twentyman et al (1980), involving alternating in vivo and in vitro passaging. $10^5$ cells were inoculated subcutaneously on the lower dorsum of mice and grown until they reached a size of 8 ± 1 mm mean diameter.

### Drugs

IdUrd (5-iodo-2′-deoxyuridine; Sigma, Zwijndrecht, The Netherlands) was dissolved in a sterile saline solution and injected intraperitoneally (i.p.) at a dose of 3, 10 or 30 mg $kg^{-1}$. Hydralazine and nicotinamide (Sigma) were dissolved in sterile saline and injected intraperitoneally (i.p.) at a dose of 5 mg $kg^{-1}$ (hydralazine) or 1000 mg $kg^{-1}$ (nicotinamide). The hydralazine dose was chosen because this dose has been shown to reduce perfusion in a murine tumour model (Trotter et al, 1989*a*). Similarly, the nicotinamide dose was chosen as that showing perfusion improvement in animal tumour models (Honess and Bleehen, 1995). Both drugs were freshly prepared before each experiment. IdUrd was injected 20 min after hydralazine or 60 min after nicotinamide. Mice were then sacrificed either 10 or 30 min after IdUrd injection. Tumours were excised and fixed in 4% buffered formaldehyde, pH 7.0, for 24 h.

IdUrd was also compared with Hoechst 33342 as a direct perfusion marker. IdUrd was injected at a dose of 30 mg $kg^{-1}$ i.p., followed 28 min later by 0.1 ml Hoechst 33342 (9 mg $ml^{-1}$), given i.v. in the tail vein. The mice were sacrificed 2 min later, the tumour immediately excised and snap-frozen in liquid nitrogen.

### Antibodies

Monoclonal antibodies were used against a surface marker on endothelial cells and against IdUrd. The first was a rat anti-mouse CD31 antibody clone MEC 13.3, dilution 1/200 (PharMingen, Leiden, The Netherlands), while the second was a mouse anti-BrdUrd, dilution 1/50 (Central Laboratory for Blood Transfusion (CLB), Amsterdam, The Netherlands). For the comparison with Hoechst 33342 on frozen sections, IdUrd labelling was visualized using a FITC-labelled goat anti-mouse antibody (Sigma) at a 1/50 dilution.

### Immunohistochemistry

Paraffin sections (4 μm) were cut and transferred to silane-coated glass slides (3-aminopropyltriethoxy silane, 2% in absolute ethanol, 5 s). Endogenous peroxidase activity was blocked with 0.3% hydrogen peroxide in methanol (30 min at RT). Microwave treatment was used for IdUrd, and 0.1% pronase for CD31, as antigen-retrieval methods. To reduce non-specific staining, Protein Block Serum-Free (undiluted, 30 min at room temperature; ITK, Uithoorn, The Netherlands) was used. After removing the protein block, the slides were incubated with either the anti-CD31 (overnight, 4°C), or the anti-BrdUrd antibodies (2 h at 37°C). A biotinylated second antibody was then added, rabbit anti-rat for CD31 (ITK) and goat anti-mouse for BRdU (Sigma), together with an avidin–biotin–peroxidase complex (Vectastain ABC Kit, Vector) for 30 min at room temperature. For the CD31 staining, an amplification step (Tyramide Signal Amplification Kit Indirect, NEN Life Science, The Netherlands) was used after incubation with the biotinylated second antibody. After washing with PBS, the peroxidase activity was detected either with a diaminobenzamide (DAB) (brown, for IdUrd) or with a DAB/nickel solution (black, for CD31) in distilled water until suitable staining developed (DAB substrate kit for peroxidase, Vector). Slides were rinsed with tap water, lightly counterstained with haematoxylin for visualization of tissue architecture, dehydrated (alcohol, xylol) and mounted with DePeX mounting medium (BDH).

For the IdUrd/Hoechst comparison, 4 μm frozen sections were cut and either stained with the anti-BrdUrd mouse antibody followed by a FITC-labelled anti-mouse second antibody, or left unstained, allowing visualization of the Hoechst dye under UV excitation.

### Flow cytometry

The staining procedure for IdUrd labelling has been published previously (Begg et al, 1988). Briefly, ethanol-fixed pieces were first incubated in a pepsin solution (0.4, 20 min, RT), incubated with a mouse anti-BrdUrd monoclonal antibody (CLB, 30 min, RT), followed by incubation with a FITC-conjugated second antibody (30 min, RT, goat anti-mouse; Sigma). For staining DNA, propidium iodide (10 mg $ml^{-1}$) plus RNase (0.2 mg $ml^{-1}$, 13.4 Kunitz units $ml^{-1}$) was used. Throughout the staining, the cells were washed between each step with diluting buffer consisting of PBS plus 0.1% bovine serum albumin and 0.05% Tween 20. All samples were analysed on a FACScan flow cytometer (Becton Dickinson) with an argon laser operating at 488 nm and 15 mW. Alignment and standardization of the instrument was performed using fluorescent beads. Emission filters were 515–545 nm Band Pass filter (green; IdUrd) and 650 nm Long Pass filter (red; DNA). 20 000 cells were analysed for each sample. The green fluorescence signal (IdUrd) was displayed on a log scale and the red fluorescence (DNA) on a linear scale.

### Image analysis

Digitized images of the CD31 and parallel IdUrd sections were processed with a macro written in Adobe Photoshop which carried out the following operations: contrast stretch to use full range of grey values, convert image from 12-bit to 8-bit to visualize image on computer monitor, sharpen image (using unsharp mask filter), duotone to make blood vessels red, or contrast enhance for IdUrd

 © 2000 Cancer Research Campaign

(black nuclei). Each image for each stain was then printed out on plastic transparent sheets using a Tektronix Phaser 240 after scaling the image to 50%. A tumour section, comprising up to 16 adjacent images captured using the microscope scanning stage, was reconstructed on the transparencies by taping adjacent images together. This was done separately for CD31 and IdUrd. The reconstructed images of the CD31-stained section were then overlayed on those for IdUrd and observed using a light box. The CD31 and IdUrd layers were then matched using two features: the form of the tumour edges and the pattern of the large vessels which were visible on both IdUrd and CD31 sections. Sometimes a match could be obtained in one area but not in another without altering the relative positions of the two reconstructed images. This could be due to folds or breaks in one of the sections, or slight differences in shrinkage during processing. In general, matching was clear, with only slight adjustments needed for the different areas. A grid, printed out on a transparency, was subsequently overlayed on the composite image to guide the scoring of vessels.

Each vessel was scored on the number of IdUrd-labelled cells in its vicinity on a scale from 0–5, 0 being no label and 5 corresponding to the maximum labelling density seen in that tumour. A vessel with labelling on one side but not on the other was scored as half of the labelling density on the positive side, e.g. label meriting a score of 4 would only be scored as 2 if occurring on one side only. The distance from a vessel in which labelling was scored was approximately equal to eight cell diameters. Each branch of a branched vessel was scored separately.

For the comparison of IdUrd and Hoechst labelling, consecutive images were captured using the computer driven microscope stage as described above. After correcting for illumination differences across each image by background subtraction, a composite image of the tumour was reconstructed. The composite image was then ‘inverted’ to convert from a dark to a light background, and from light objects (IdUdR labelled cells or Hoechst fluorescence), to dark-labelled objects.

### RESULTS

### Flow cytometry

To investigate the effect of blood-flow manipulation on IdUrd labelling, mice with subcutaneous RIF1 tumours were injected with hydralazine, nicotinamide or physiological saline and 20–60 min later injected with IdUrd. Three separate experiments were carried out, investigating IdUrd dosage, time between IdUrd injection and sacrifice, and time between hydralazine and IdUrd. Results of the first experiment investigating different IdUrd doses of 3–30 mg $kg^{-1}$ employing an IdUrd-sacrifice time of 30 min are shown in Figure 1. In control mice, neither labelling index (LI) nor labelling intensity (fluorescence ratio, FR) changed significantly with increasing IdUrd doses (Figure 1 top and middle panels, left bars). Hydralazine appeared to cause decreases in both LI and FR, which was most obvious at the lowest IdUrd dose. However, these changes were not statistically significant ($P > 0.05$, *t*-test). In this experiment there was a 60 min interval between hydralazine and IdUrd, which was reduced in subsequent experiments (see below). Variation in fluorescence intensity of IdUrd labelling for each tumour, expressed as the coefficient of variation (CV) of green fluorescence, was also investigated. The CV was decreased after nicotinamide, although not significantly (*t*-test). Hydralazine had little effect.

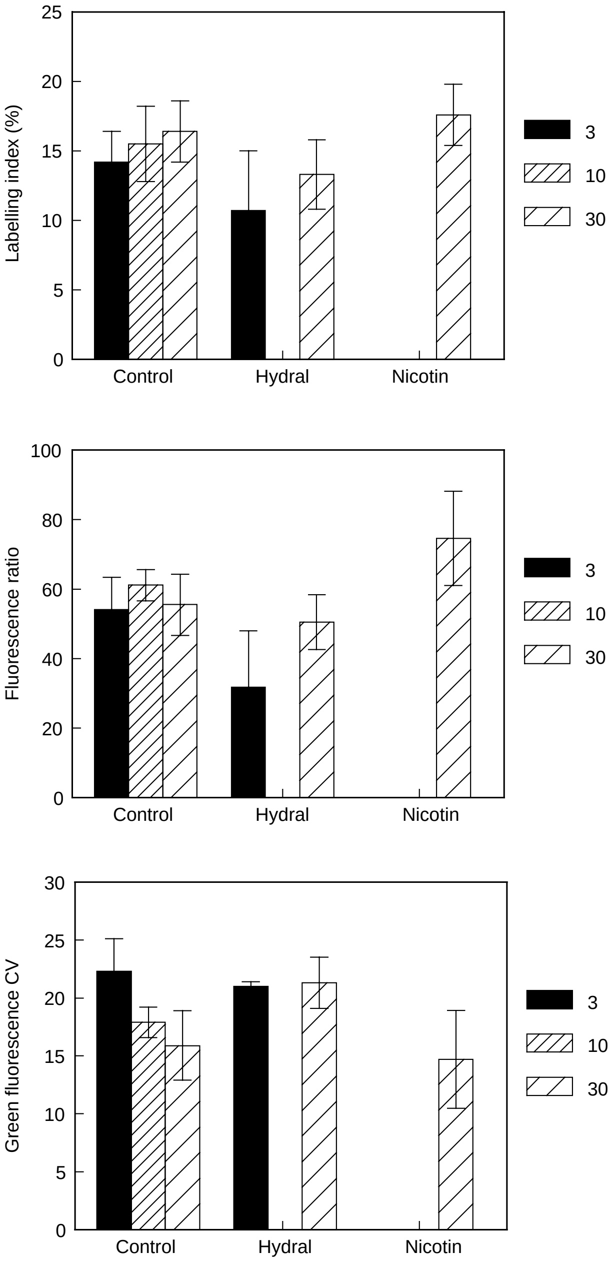

**Figure 1** IdUrd labelling index, labelling intensity (fluorescence ratio labelled : unlabelled cells) and variation in labelling intensity per tumour (CV) of RIF1 tumours for control mice or mice treated with hydralazine to reduce perfusion or nicotinamide to increase it. Mice were given 3, 10 or 30 mg $kg^{-1}$ IdUrd. Errors are ± SD, three mice per group. Some combinations were not done, namely: 10 mg $kg^{-1}$ IdUrd for both hydralazine and nicotinamide and 3 mg $kg^{-1}$ for nicotinamide

In the second experiment, we investigated lower IdUrd doses down to 0.3 mg kg–1 and shorter times between IdUrd and sacrifice, following the reasoning that differences dependent on blood-flow may be more evident during the early uptake phase and at non-saturating IdUrd concentrations. Using a 10 min IdUrd-sacrifice interval (Figure 2, top panels), both LI and FR increased in control mice with increasing IdUrd dose from 0.3–3 mg kg–1. The interval between hydralazine and IdUrd was decreased from

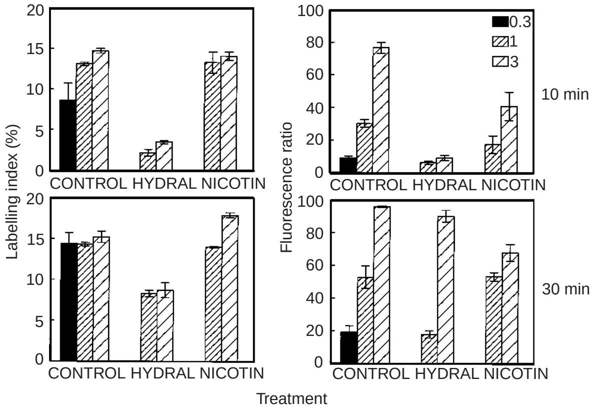

**Figure 2** IdUrd labelling index and labelling intensity of RIF1 tumours in control mice or mice treated with hydralazine to reduce perfusion or nicotinamide to increase it. Mice were given 0.3, 1 or 3 mg kg$^{-1}$ IdUrd (see legend upper right) and sacrificed 10 min (upper panels) or 30 min (lower panels) after IdUrd. Errors represent range, two mice per group

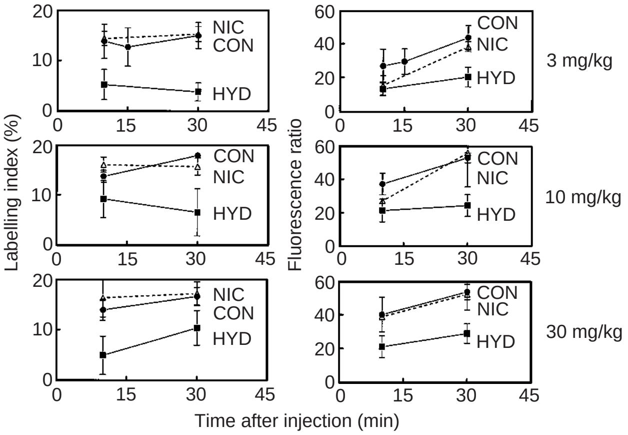

**Figure 3** IdUrd labelling index (left) and intensity (right) of RIF1 tumours in control mice or mice treated with hydralazine or nicotinamide. Mice were given 3, 10 or 30 mg kg$^{-1}$ IdUrd and sacrificed 10 or 30 min after IdUrd. Errors are ± SD, 3–4 mice per group

60–20 min, based on optimal blood-flow reduction data in mouse tumours reported in the literature (Trotter et al, 1989a). Hydralazine clearly reduced both numbers and intensities of labelled cells. No obvious increase in either LI or FR was evident after nicotinamide. At the 30 min sacrifice time, LI remained constant with dose, but FR increased approximately proportionally to IdUrd dose. Suppressive effects of hydralazine were somewhat less evident than in the 10 min data. Nicotinamide caused no increase in labelling, or even a slight decrease.

The protocol for the third experiment followed the first, but used an optimum hydralazine-IdUrd interval of 20 min and did not exclude any group combinations. The results (Figure 3) confirmed the data of the first two experiments in showing a distinct suppressive effect of hydralazine and little effect of nicotinamide. Pooling times and doses for this last more extensive experiment, the mean LIs for the control, nicotinamide and hydralazine groups were 15.0 ± 2.5, 16.3 ± 2.1, and 6.7 ± 3.8 respectively. The mean FRs for the control, nicotinamide and hydralazine groups were 42.0 ± 11.2, 40.0 ± 17.4, and 21.7 ± 7.1 respectively (errors ± 1 SD, $n$ = 18–19). For both LI and FR, the difference between control hydralazine groups was significant ($P < 0.001$).

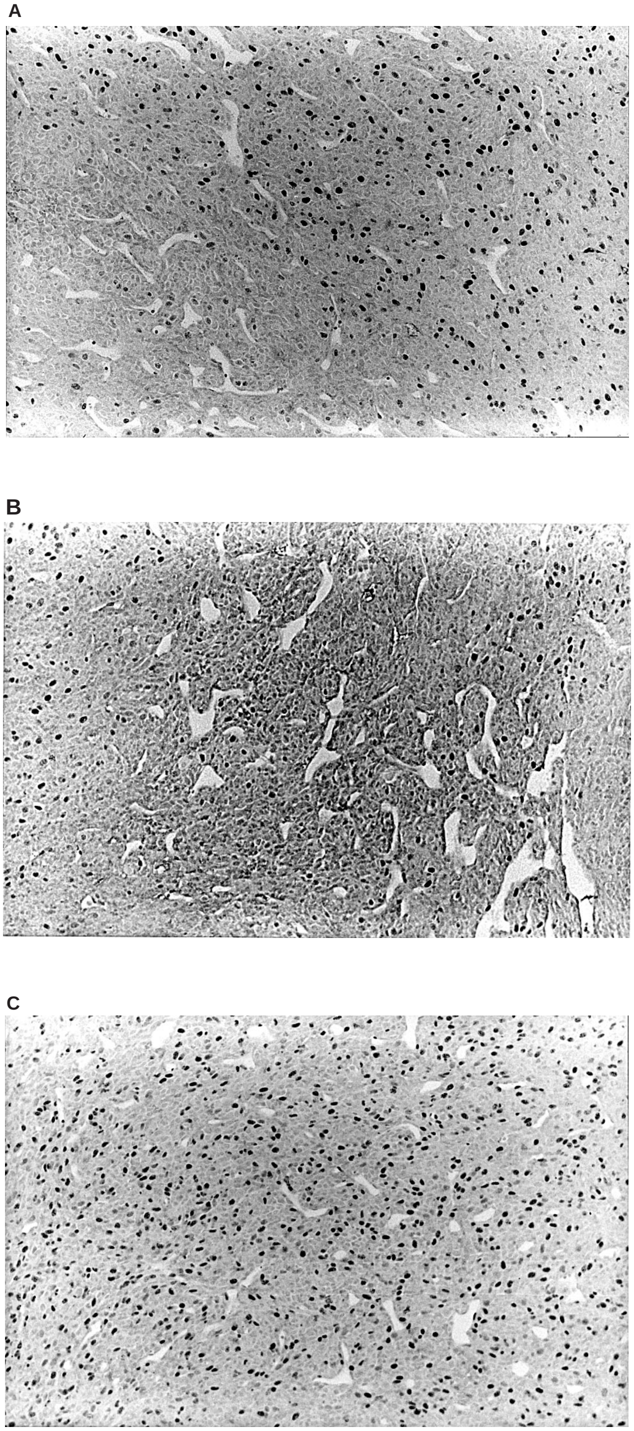

**Figure 4** Examples of images of immunohistochemical labelling with IdUrd in control RIF1 tumours (**A**) or in those treated with hydralazine (**B**) or nicotinamide (**C**) (magnification × 25). Decreased labelling after hydralazine and a tendency for more uniform labelling after nicotinamide is evident

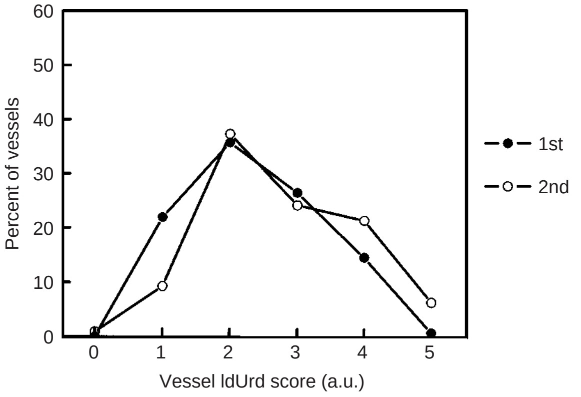

**Figure 5** Reproducibility of IdUrd scoring around vessels. The same microscope fields in RIF1 tumour sections were scored on two occasions separated by several weeks by the same observers

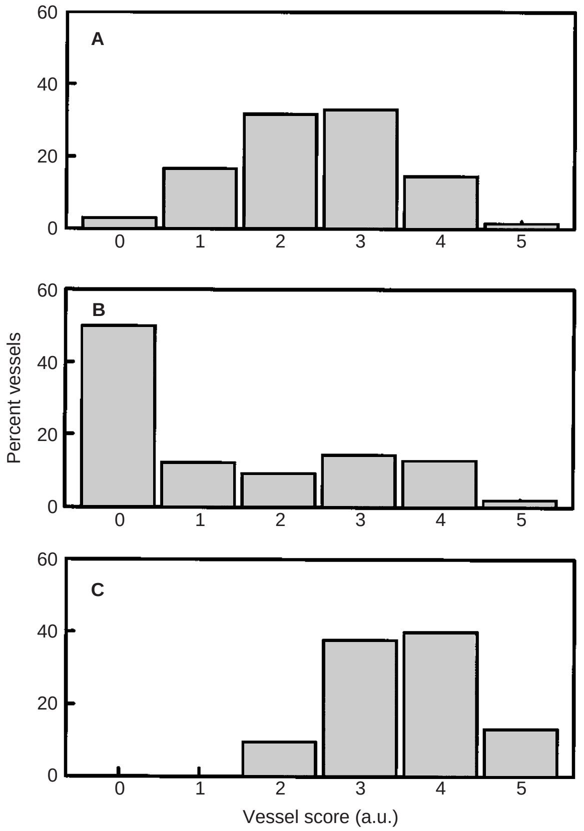

**Figure 6** Histograms of IdUrd labelling around vessels for control RIF1 tumours (**A**) or tumours treated with hydralazine (**B**) or nicotinamide (**C**). A clear increase in low IdUrd scores is seen after hydralazine and a decrease after nicotinamide. The number of vessels scored were 733, 633 and 274 for control, hydralazine and nicotinamide respectively

### Image analysis

Images of immunohistochemically stained slides for IdUrd and vasculature were digitized and analysed for labelling variations around individual vessels. IdUrd labelling patterns showed some heterogeneity in control tumours (Figure 4A) which appeared to become more homogeneous after nicotinamide treatment (Figure 4C). After hydralazine, labelling was clearly reduced (Figure 4B).

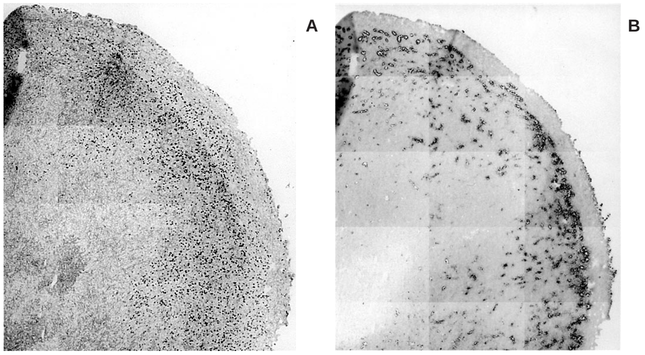

**Figure 7** Comparison of IdUrd labelling (**A**) with Hoechst 3342 perfusion (**B**) from adjacent frozen sections of a RIF1 tumour. Each section is a composite of contiguous images captured using a $\times$ 10 objective. Similar labelling patterns are evident for both labels

To quantify labelling, each vessel was scored on a scale of 0–5, indicating no–high labelling, and was achieved by matching adjacent sections of vascular and IdUrd staining (see Methods). Histograms of the scores showed an approximate normal distribution, with some variation from area to area within control tumours. Of note was the fact that significant but small numbers of vessels were observed with no label in their vicinity.

In order to assess reproducibility of scoring, one area of the tumour was rescored approximately 4 weeks after the first scoring. Although there were minor differences (Figure 5), the reproducibility was good. In the first scoring session, 109 vessels were noted and scored, while in the second, 107 were found. This was also regarded as good agreement.

Next and most importantly, a comparison of tumours was made under conditions of reduced or increased perfusion (Figure 6). Mice from the experiment shown in Figure 3 were used, which received an IdUrd dose of 30 mg $kg^{-1}$ and which were sacrificed 30 min later. After hydralazine (Figure 6B), there was a large increase in the fraction of vessels with no surrounding IdUrd labelling, reaching a value close to 50% compared with 3% of vessels in control tumours. Nicotinamide showed a shift to the right compared with control tumours, and no vessels were found together with a complete absence of IdUrd labelling, indicating better perfusion after administration of this drug. The median values of the vessel scores were 2.0, 0.5 and 3.5 for control, hydralazine and nicotinamide respectively.

The approach was further validated by directly comparing IdUrd labelling patterns with those after Hoechst injection, a direct perfusion marker. Adjacent sections were either stained with an anti-IdUrd antibody or left unstained, allowing visualization of the blue fluorescent Hoechst dye under UV excitation. A series of consecutive images was then captured using a computer-controlled stepping microscope stage and a $\times$ 10 objective. Figure 7 shows tumour sections reconstructed from the separate images. The IdUrd staining (Figure 7A) shows considerable heterogeneity of labelling, with little or no label near the tumour centre. The Hoechst images (Figure 7B) show perfused blood vessels mainly on the periphery of the tumour, with little visible perfusion in the tumour centre. The similarity of these patterns is consistent with the hypothesis that IdUrd labelling can monitor tumour perfusion.

### DISCUSSION

The present data show that changes in vascular perfusion in this mouse tumour model are reflected by changes in labelling of the

*© 2000 Cancer Research Campaign*

thymidine analogue IdUrd. This was found by both flow cytometry, where the whole tumour is investigated but where geographical information is lost, and by image analysis, where individual vessels can be studied. The most clear effects were seen with hydralazine, in which reductions in both numbers and intensities of labelling were observed, and where the number of vessels with no surrounding IdUrd labelling increased dramatically. This is consistent with the study of Trotter and colleagues (1989*a*) who showed a large increase in vessels showing no perfusion after hydralazine treatment in the SCCVII murine tumour using a double fluorescent marker staining technique.

Nicotinamide showed less obvious effects with flow cytometry, although image analysis showed fewer vessels with no/low surrounding IdUrd, indicating an improvement in perfusion. The RIF1 tumour is relatively well perfused in untreated animals, as evidenced from the low proportion (3%) of vessels with no label, although intratumoural perfusion variations have been reported for this tumour (van Geel et al, 1996). Blood-flow studies have shown little effect of nicotinamide in RIF1 tumours (Honess and Bleehen, 1993; 1995), although nicotinamide alone at the dose used here has been shown to sensitize the hypoxic tail of the single-dose radiation survival curve, indicating perfusion and oxygenation improvements (Horsman et al, 1987). The studies are consistent with little increase in total blood-flow but a redistribution after nicotinamide, leading to fewer closed or low-flow vessels and a subsequent reduction in the hypoxic fraction. This was seen in our study by a right shift of the IdUrd labelling histogram around vessels.

The correspondence between IdUrd supply and labelling intensity will depend on the IdUrd dose-range, and from the present data is apparently linear only at doses less than 3 mg $kg^{-1}$. The labelling index, however, remained remarkably constant over the wide dose-range of 0.3–30 mg $kg^{-1}$. The drop in LI after hydralazine with an IdUrd dose of 30 mg $kg^{-1}$ therefore implies a perfusion decrease of a factor of over 100, and probably a total shut-down in many vessels, consistent with the Trotter study (Trotter et al, 1989*a*). This also implies that perfusion decreases could be detected earlier from labelling intensity than from labelling index changes. If the IdUrd supply is only temporarily decreased, the cells will probably still express proteins responsible for, or associated with, cell-cycle progression, such as PCNA, cyclin A and Ki67. A refinement of the proposed technique would therefore be to double-stain for IdUrd and an endogenous proliferation marker IdUrd-negative but Ki67-positive regions would indicate a temporary perfusion decrease.

IdUrd labelling may not always mirror perfusion. Firstly, in a cluster of vessels in close proximity, one vessel could be poorly or non-perfused but still have IdUrd labelling around it through supply from neighbouring vessels. This is unlikely to be a problem when making the link with hypoxia, since oxygen will probably also be supplied from the surrounding vessels, preventing local hypoxia. Lack of labelling around a vessel could also occur if there were proliferating cells but no S-phase cells in the vicinity of a vessel. This is statistically unlikely with LIs typically found in many human tumours. Thirdly, resumption of flow in a vessel closed for sufficient time to halt proliferation of tumour cells around it would supply IdUrd but no labelling would occur. The frequency of such an occurrence is not known. Lastly, not all vessels will be stained, as judged from areas showing considerable IdUrd labelling but no visible vessels. Whether these missed, and therefore unscored, vessels show a different range of perfusion than the stained vessels remains to be determined.

The scoring of IdUrd labelling around vessels in the manner described here has a subjective element and is semi-quantitative. Several factors influence the scoring including, first and foremost, the definition of a vessel. In this study, we scored all branches of a complex structure as separate vessels, based on the observation that labelling around branches was often less than around the main vessel. Secondly, to define the maximum scoring we scanned the tumour and picked out areas of maximum labelling which were assigned the score 5. This would vary between tumours, and so remains a relative scale. The scoring will be most accurate in high-LI tumours and least accurate in low-LI tumours. The mean LI in the present tumour was around 15%. Human head and neck tumours typically have an LI around 12% (Begg et al, 1999), making such scoring feasible on this type of human tumour. Thirdly, the distance from a vessel included in the score was 5–6 cell diameters, based on observations on labelling around relatively isolated vessels. Despite the subjective element, the method proved surprisingly robust in the reproducibility test. The development of a suitable double-staining protocol in which vessels and IdUrd could be stained with different colours in the same section would avoid the necessity of matching and thus improve and speed up the technique.

In conclusion, the use of an exogenous marker originally designed for cell kinetic studies appears to provide additional information reflecting local perfusion variations. As such, it could provide a useful supplement to methods investigating human tumour hypoxia. Several centres, including our own, are combining hypoxia measurement techniques, e.g. administration of bioreductive marker drugs, with I- or Br-dUrd, in order to additionally measure cell kinetic parameters. Application of the technique described here would also allow estimates of perfusion-limited hypoxia from the fraction of vessels with little or no analogue labelling around them.

### ACKNOWLEDGEMENTS

We would like to thank Erwin Bellon (Laboratory for Medical Imaging Research, Katholieke Universiteit Leuven, Belgium) for his willing help and expertise in making the composite Hoechst and IdUrd images. We also thank Hugo Oppelaar for his help with some of the mouse experiments. We also thank Harry Bartelink for continued support and encouragement. This study was funded in part by NIH grant 1R1CA80146.

### REFERENCES

Begg AC, Moonen L, Hofland I, Dessing M and Bartelink H (1988) Human tumour cell kinetics using a monoclonal antibody against iododeoxyuridine: intratumour sampling variations (published erratum appears in *Radiother Oncol* **15**: 215). *Radiother Oncol* **11**: 337–347

Begg AC (1995) The clinical status of Tpot as a predictor? Or why no tempest in the Tpot. *Int J Radiat Oncol Biol Phys* **32**: 1539–1541

Begg AC, Haustermans KM, Hart AA, Dische S, Saunders MI, Zackrisson B, Gustafsson H, Coucke P, Paschoud N, Hoyer M, Overgaard J, Antognoni P, Richetti A, Bourhis J, Bartelink H, Horiot JC, Corvo R, Giaretti W, Awwad HK and Shouman T (1999) The value of pretreatment cell kinetic parameters as predictors for radiotherapy outcome in head and neck cancer: a multicenter analysis. *Radiother Oncol* **50**: 13–23

Bernsen HJ, Rijken PF, Oostendorp T and van der Kogel AJ (1995) Vascularity and perfusion of human gliomas xenografted in the athymic nude mouse. *Br J Cancer* **71**: 721–726

*© 2000 Cancer Research Campaign*

Bollinger A (1993) Microlymphatics of human skin. *Int J Microcirc Clin Exp* **12**: 1–15

Brown JM (1979) Evidence for acutely hypoxic cells in mouse tumours and a possible mechanism of reoxygenation. *Br J Radiol* **52**: 650–656

Chaplin DJ, Durand RE and Olive PL (1986) Acute hypoxia in tumours: implications for modifiers of radiation effects. *Int J Radiat Oncol Biol Phys* **12**: 1279–1282

Chaplin DJ, Olive PL and Durand RE (1987) Intermittent blood flow in a murine tumour: radiobiological effects. *Cancer Res* **47**: 597–601

Dewhirst MW (1998) Concepts of oxygen transport at the microcirculatory level. *Semin Radiat Oncol* **8**: 143–150

Durand RE (1983) Oxygen enhancement ratio in V79 spheroids. *Radiat Res* **96**: 322–334

Honess DJ and Bleehen NM (1993) Effects of the radiosensitising agent nicotinamide on relative tissue perfusion and kidney function in C3H mice. *Radiother Oncol* **27**: 140–148

Honess DJ and Bleehen NM (1995) Perfusion changes in the RIF-1 tumour and normal tissues after carbogen and nicotinamide, individually and combined. *Br J Cancer* **71**: 1175–1180

Horsman MR, Chaplin DJ and Brown JM (1987) Radiosensitization by nicotinamide in vivo: a greater enhancement of tumour damage compared to that of normal tissues. *Radiat Res* **109**: 479–489

Huber M, Franzeck UK and Bollinger A (1984) Permeability of superficial lymphatic capillaries in human skin to FITC-labelled dextrans 40 000 and 150 000. *Int J Microcirc Clin Exp* **3**: 59–69

Kimura H, Braun RD, Ong ET, Hsu R, Secomb TW, Papahadjopoulos D, Hong K and Dewhirst MW (1996) Fluctuations in red cell flux in tumour microvessels can lead to transient hypoxia and reoxygenation in tumour parenchyma. *Cancer Res* **56**: 5522–5528

Kotelnikov V, Cass L, Coon JS, Spaulding D and Preisler HD (1997) Accuracy of histone H3 messenger RNA in situ hybridization for the assessment of cell proliferation in human tissues. *Clin Cancer Res* **3**: 669–673

Reinhold HS and van den Berg-Blok A (1983) Vascularization of experimental tumours. *Ciba Found Symp* **100**: 100–119

Spiro IJ, Rice GC, Durand RE, Stickler R and Ling CC (1984) Cell killing, radiosensitization and cell cycle redistribution induced by chronic hypoxia. *Int J Radiat Oncol Biol Phys* **10**: 1275–1280

Sutherland RM and Franko AJ (1980) On the nature of the radiobiologically hypoxic fraction in tumours. *Int J Radiat Oncol Biol Phys* **6**: 117–120

Thomlinson RH and Gray LH (1955) The histological structure of some human lung cancers as the possible implications for radiotherapy. *Brit J Cancer* **9**: 539

Trotter MJ, Acker BD and Chaplin DJ (1989*a*) Histological evidence for nonperfused vasculature in a murine tumour following hydralazine administration. *Int J Radiat Oncol Biol Phys* **17**: 785–789

Trotter MJ, Chaplin DJ, Durand RE and Olive PL (1989*b*) The use of fluorescent probes to identify regions of transient perfusion in murine tumours. *Int J Radiat Oncol Biol Phys* **16**: 931–934

Twentyman PR, Brown JM, Gray JW, Franko AJ, Scoles MA and Kallman RF (1980) A new mouse tumour model system (RIF-1) for comparison of end-point studies. *J Natl Cancer Inst* **64**: 595–604

van Geel IP, Oppelaar H, Rijken PF, Bernsen HJ, Hagemeier NE, van der Kogel AJ, Hodgkiss RJ and Stewart FA (1996) Vascular perfusion and hypoxic areas in RIF-1 tumours after photodynamic therapy. *Br J Cancer* **73**: 288–293

*© 2000 Cancer Research Campaign*
